# Ice-dependent winter survival of juvenile Atlantic salmon

**DOI:** 10.1002/ece3.481

**Published:** 2013-01-24

**Authors:** R D Hedger, T F Næsje, P Fiske, O Ugedal, A G Finstad, E B Thorstad

**Affiliations:** Norwegian Institute for Nature ResearchP.B. 5685 Sluppen, NO-7584, Trondheim, Norway

**Keywords:** Apparent survival, energy content, ice cover, *Salmo salar*, size-dependent mortality

## Abstract

Changes in snow and ice conditions are some of the most distinctive impacts of global warming in cold temperate and Arctic regions, altering the environment during a critical period for survival for most animals. Laboratories studies have suggested that reduced ice cover may reduce the survival of stream dwelling fishes in Northern environments. This, however, has not been empirically investigated in natural populations in large rivers. Here, we examine how the winter survival of juvenile Atlantic salmon in a large natural river, the River Alta (Norway, 70°N), is affected by the presence or absence of surface ice. Apparent survival rates for size classes corresponding to parr and presmolts were estimated using capture-mark-recapture and Cormack-Jolly-Seber models for an ice-covered and an ice-free site. Apparent survival (Φ) in the ice-covered site was greater than in the ice-free site, but did not depend on size class (0.64 for both parr and presmolt). In contrast, apparent survival in the ice-free site was lower for larger individuals (0.33) than smaller individuals (0.45). The over-winter decline in storage energy was greater for the ice-free site than the ice-covered site, suggesting that environmental conditions in the ice-free site caused a strong depletion in energy reserves likely affecting survival. Our findings highlight the importance of surface ice for the winter survival of juvenile fish, thus, underpinning that climate change, by reducing ice cover, may have a negative effect on the survival of fish adapted to ice-covered habitats during winter.

## Introduction

In seasonal temperate environments, changes in snow and ice conditions are one of the most distinct impacts of global warming (Smol et al. 2005; Kausrud et al. 2008). Due to reduced resource levels and harsh climatic conditions, the winter is usually a critical period with regard to survival (Hurst 2007; Mcnamara and Houston 2008), and nonmigratory animals often face physiological constraints and negative energy budgets. In order to survive, such animals therefore rely on various strategies based on combinations of stored energy reserves and foraging (Bull et al. 1996; Finstad et al. 2010). Ice-formation and deep snow reduces access to food (Solberg et al. 2001; Doherty and Grubb 2002; Robinson et al. 2007), but may also function as a shield from predators (Finstad et al. 2004b; Berg et al. 2008).

For cold temperate and Arctic lakes and rivers, surface ice in combination with snow cover creates a major contrast in habitat characteristics to summer, particularly with regard to light. Salmonids have been previously shown to be sensitive to changes in surface ice conditions (Finstad et al. 2004a; Finstad and Forseth 2006; Helland et al. 2011). First, surface ice may cause behavioral changes, lowering metabolic activity and increasing energy intake, which in turn may reduce the depletion of energy stores (Finstad et al. 2004a,b). This effect may be particularly strong in northern populations that experience months of ice cover (Finstad and Forseth 2006). Secondly, surface ice may reduce risk from endotherm predators (Valdimarsson and Metcalfe 1998). Finally, surface ice may prevent the formation of anchor ice (Power et al. 1993; Roussel et al. 2004). Both historical time-series and future scenario modeling emphasize reduction in ice cover in Northern hemisphere watersheds as one of the major responses to global warming (Magnuson et al. 2000; Dibike et al. 2011). This reduction in ice cover may impact on salmonids by increasing metabolic activity and reducing energy intake, thereby causing a depletion of energy stores. Additionally, risk from predators may increase, and anchor ice may be able to form, which may obstruct access to interstitial spaces in the substrate that juveniles use for shelter. It is therefore conceivable that winter survival may be reduced as a result of global warming.

**Figure 1:**
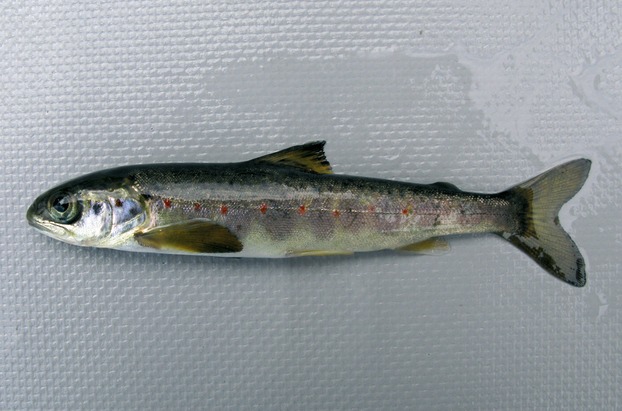
A wild Atlantic salmon smolt. Total length is ∼130 mm (Photo: Eva B. Thorstad).

Atlantic salmon (Fig 1) is native to the North Atlantic Ocean and adjoining regions. Due to a complex life cycle, and its value as a recreational and commercial resource of high conservation relevance, Atlantic salmon is also a species under considerable focus with regard to effects of future global warming (Jonsson and Jonsson 2009, 2011). Juveniles typically rear for 1–8 years in fresh water before undertaking long marine migrations, and remain longer in fresh water in northern than southern populations making these populations particularly vulnerable to altered surface ice conditions (Jonsson and Jonsson 2009, 2011).

Despite the considerable focus of current literature dealing with climate change related effects on salmonid juveniles, empirical studies have almost exclusively been carried out in small rivers and streams (Huusko et al. 2007). Due to the presumed highly context-dependent nature of winter survival patterns, it is an open question if results from small streams and rivers can be transferred to the main stem of large rivers, holding the important rearing habitats of Atlantic salmon. Here, we utilize a field study on a large river (River Alta, Norway) where hydropower regulation has created natural gradients in ice cover to examine large scale patterns of winter survival. Apparent survival probability was estimated in two sites, one site ice-covered during winter and the other relatively ice-free during winter as a result of proximity to a hydropower reservoir, using a capture-mark-recapture (CMR) program. It was hypothesized that winter survival in the ice-free site would be less than that in the ice-covered site, and that winter-survival of parr would be less than that of pre-smolts.

## Materials and Methods

### Study area

**Figure 2:**
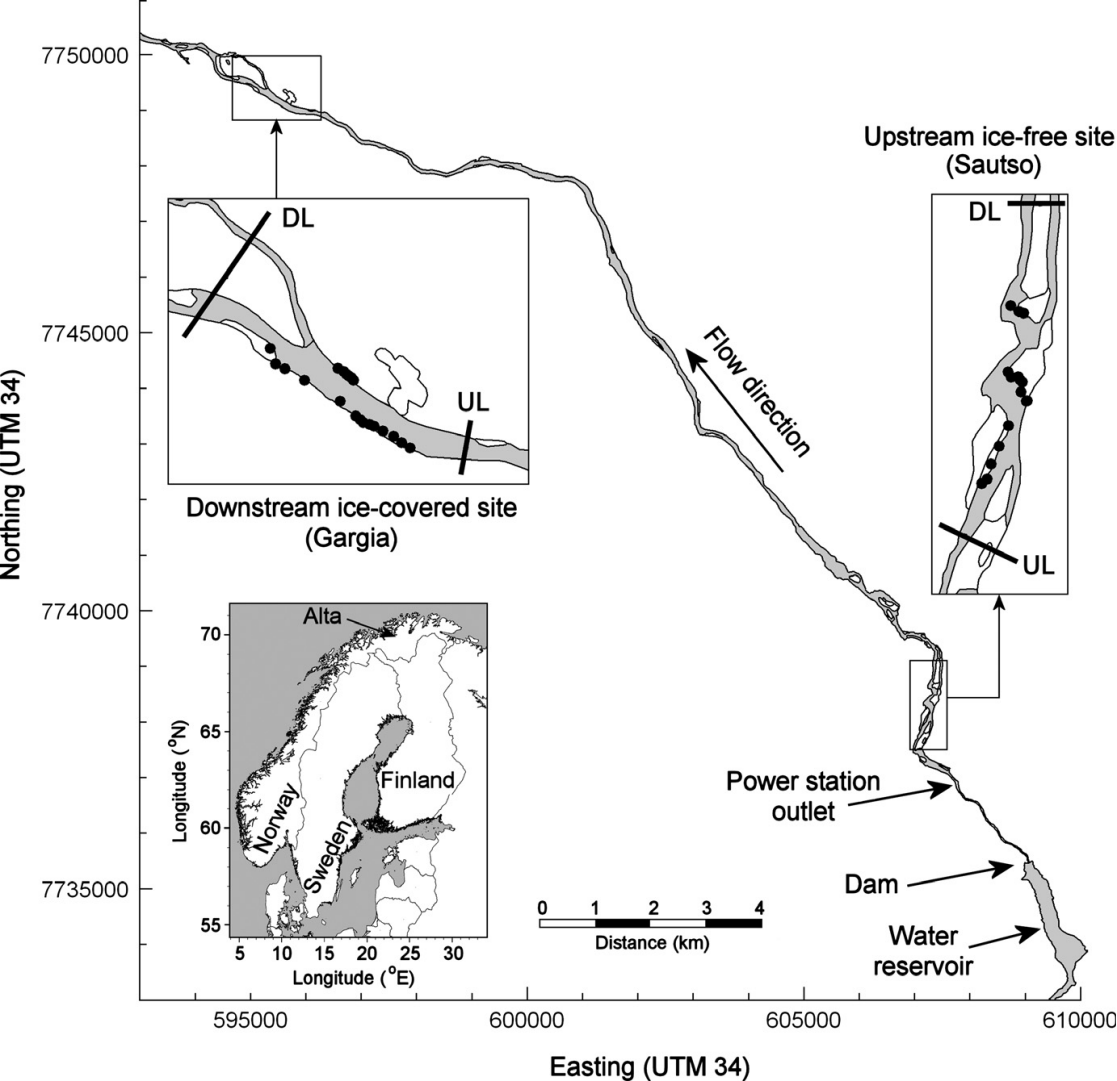
The River Alta, showing the two study sites: Gargia (downstream, distant from the hydropower reservoir, and ice-covered over winter) and Sautso (upstream, near to the hydropower reservoir and relatively ice-free over winter). Positions of Atlantic salmon release are shown by filled circles. Upstream (UL) and downstream (DL) limits of the recapture zone are also shown.

The River Alta (70^o^N, 23^o^E, catchment area of 7389 km^2^, length of main stem 160 km, mean annual water discharge of 75 m^3^s^−1^) is an Atlantic salmon river in Northern Norway (Jensen 2003) (Fig 2). The downstream 10 km of the river has a fine substratum, whereas the more upstream part of the river consists of pools or faster flowing reaches with coarser substrates. The river was regulated for hydropower production in 1987, with the creation of a hydropower reservoir 50 km upstream from the river mouth. The outlet of the water tunnel from the hydropower plant is located at the upper end of the salmon producing section, 2.5 km downstream of the dam. The mean annual winter discharge (December to April) has increased from 14 m^3^s^−1^ (1972–1986) to 29 m^3^s^−1^ (1988–2003) (Ugedal et al. 2008). Winter water temperature immediately downstream of the reservoir has increased by 0.2–0.4°C since creation of the reservoir, which may prevent the formation of surface ice in the 4–5 km stretch downstream of the power plant outlet (Ugedal et al. 2008). Surface ice is present further downstream throughout winter. Reported light levels under ice in this river range between <0.01 μmol s^−1^ m^−2^ and 6.3 μmol s^−1^ m^−2^, with daily averages similar to night-time in winter due to the polar night (Finstad et al. 2004a). Atlantic salmon is the dominating species in the River Alta. Brown trout (*Salmo trutta*) is also commonly caught. In addition, Arctic char (*Salvelinus alpinus*) is most common in the lower part, whereas whitefish (*Coregonus lavaretus*), European grayling (*Thymallus thymallus*) and minnow (*Phoxinus phoxinus*) are most common in the upper part. Flounder (*Platichthys flesus*), three-spined stickleback (*Gasterosterus aculeatus*), pike (*Esox lucius*), burbot (*Lota lota*), yellow perch (*Perca fluviatilis*) and eel (*Anguilla anguilla*) are occasionally found.

Juvenile Atlantic salmon density in the upstream part of the reach accessed by Atlantic salmon decreased after the river was developed for hydropower production, but in recent years, there has been a partial recovery (Ugedal et al. 2008). In contrast, there has been a consistent increase in juvenile Atlantic salmon abundance in the middle and downstream section, likely due to the increased and stable water discharge during winter.

Two study sites were selected: (1) a site that was ice-covered during winter (Gargia), ∼20 km distant from the hydropower reservoir; and (2) a site that was relatively ice-free during winter (Sautso), just ∼2 km downstream of the hydropower plant outlet (Fig 2). At Sautso, parts of the study area were covered by border ice during the study period, but the river was mainly open in the middle of the channel during winter. At Gargia, ice covered the entire river during most of the winter after freeze-up around the middle of December. These sites were selected from analysis of previous habitat surveys (Økland et al. 2004) with the criteria that (1) they provided suitable habitat for salmon to reside over winter and (2) they differed little between themselves in terms of channel habitat characteristics (velocity, depth and substrate size), reducing channel habitat-induced bias on capture probability and survival. The principle substrate class in both sites was cobbles (8–35 cm). Most areas of both sites had depths ranging between 0.2 and 0.8 m. Current velocity was not measured during electrofishing. However, previous measurements at a discharge of 40 m^3^s^−1^ have shown ∼95% of the channel at both sites having surface current velocities varying between 0.2 and 1 ms^−1^. Channel widths of the sites were varying, but similar (mean widths ∼60 m).

**Figure 3:**
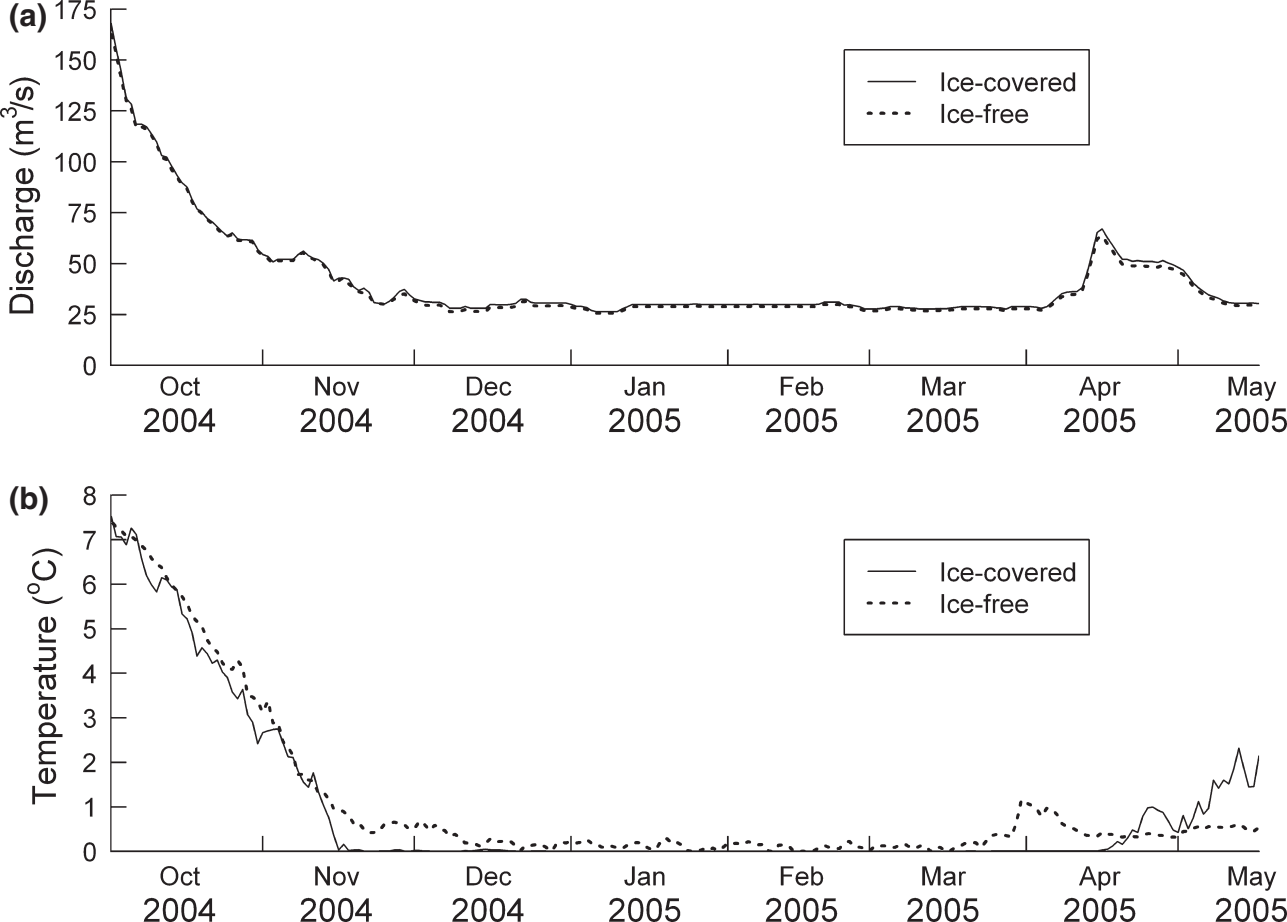
Average daily (a) water discharge (m^3^s^−1^) and (b) water temperature (°C) from 1 October 2004 to 15 May 2005 at the ice-covered and ice-free sites in the River Alta. Data were from the Norwegian Water Resources and Energy Directorate.

Water temperatures and discharges were measured at both study sites (Fig 3). Water temperatures at the ice-free site were higher than those at the ice-covered site, both prior to and during ice cover. Over-winter (December to mid-April) temperatures were lower at the ice-covered site (mean = 0.00°C) than the ice-free site (mean = 0.23°C). Over-winter discharges were very similar at the two sites (ice-covered, mean 30 m^3^s^−1^; ice-free, mean 29 m^3^s^−1^).

### Capture-mark-recapture

A capture-mark recapture (CMR) study was conducted to provide data for fitting Cormack-Jolly-Seber (CJS) models for estimating the survival over the winter of 2004–2005. Each site, of approximately 700 m in river length, was compartmentalized longitudinally into stations of 25 m in length, and individuals were then captured within each section by electrofishing along the river banks during mid-October 2004. Electrofishing was conducted by wading along one of the banks using backpack mounted Geomega FA 4 apparatus (Terik Technology). The bank that was selected was chosen as that which had most optimal conditions for electrofishing and the electrofishing switched banks when poor conditions (such as deep areas) were encountered. Approximately, 20–25% of the channel was electrofished. Discharge was rather high during the capture (decreasing from 110 to 80 m^3^s^−1^) and wetted width approached the channel width of ∼60 m. Individuals were captured in 21 stations in the downstream ice-covered site and 16 stations in the upstream ice-free site. Each captured individual above 60 mm total length was classified as a parr or a presmolt according to size (parr total length <105 mm; presmolt total length ≥105 mm), based on the minimum observed length of smolting fish caught in a smolt trap (situated 11 km upstream from the river mouth) from another study (see Ugedal et al. 2008; Davidsen et al. 2009). The age of the fish at tagging ranged from 1 + to 4 + according to aging of recaptured fish in spring.

Captured fish were anesthetised with Metomidate and measured (total length) to the nearest mm and weighed to the nearest 0.1 g. A scalpel was used to make a small incision in the fish abdomen, and a Passive Integrated Transponder (HDX PIT, 12 mm long × 2 mm diameter, ISO 11784/85, frequency 134.2 KHz, provided by Trac ID Systems) was gently pushed into the abdominal cavity through the incision (cf. Gries and Letcher 2002). The fish was allowed to recover, and was then released at the center of the 25 m station where it had been captured. The geographical position was recorded for each station with a GPS. In total, 1441 and 1226 individuals were released in the ice-covered and ice-free sites, respectively (Table 1). Mean total length of released parr was 85 mm (min = 59 mm, max = 104 mm) and presmolts was 121 mm (min = 105 mm, max = 165 mm) in the ice-covered site. Mean total length of released parr was 86 mm (min = 60 mm, max = 104 mm) and presmolts was 125 mm (min = 105 mm, max = 170 mm), respectively, in the ice-free site. Body masses were similar between the two sites: parr, mean = 5.3 g (1.6–18.6 g) in the ice-free site and 5.6 g (1.9–13.1 g) in the ice-covered site; presmolt, mean = 14.7 g (6.0–13.6 g) in the ice-free site and 17.6 g (2.9–51.2 g) in the ice covered site.

**Table 1:** Dates of PIT- tagging and recapture surveys of Atlantic salmon juveniles in the River Alta

Site	Tagging and release	First recapture survey	Second recapture survey
Ice-covered	9–18 October 2004 (694 parr, 747 presmolt)	1–7 May 2005 (212 parr, 214 presmolt)	9–14 May 2005 (150 parr, 150 presmolt)
Ice-free	13–16 October 2004 (486 parr, 740 presmolt)	1–6 April 2005 (84 parr, 78 presmolt)	19–25 April 2005 (32 parr, 32 presmolt)

Recapture was conducted in two periods early in the following spring to avoid the effect of flooding, but was delayed in the downstream site because of the presence of surface ice (Table 1). The length of the river electrofished for recapture extended 200 m upstream and 400 m downstream from the area where the fish were released to reduce bias caused by fish emigrating from the release area, and to study possible emigration from the tagging area. The river width covered by electrofishing during recapture varied from 3 to 50 m (mean = 15 m) at the ice-covered site and 3–50 m (mean = 12 m) at the ice-free site. Total area covered by the recapture events was approximately 19 000 m^2^ in both areas.

At the first recapture event, the fish were anesthetized with clove-oil, examined for the presence of a PIT-tag with a hand-held detector, measured and weighed and after recovery released at the center of the section where they had been captured. At the second recapture event, PIT-tagged fish were sacrificed for further analysis of energetic content. The second recapture event in spring allow us to obtain separate estimates for apparent survival and apparent encounter between tagging in October and the first recapture event in spring (see further details below).

At the first recapture event in the ice-free site (1–6 April), the mean water discharge was 29 m^3^s^−1^ and the mean water temperature was 0.9°C. The first recapture event in the ice-covered site took place a month later (1–7 May) at a somewhat higher discharge (mean 39 m^3^s^−1^), but the mean water temperature was 0.9°C, which was similar to the temperature at the ice-free site at first recapture.

Cormack-Jolly-Seber (CJS) models were used to estimate (1) the apparent survival probability (Φ), which is the probability that an individual will both survive and remain within the study area, and (2) the apparent encounter probability (*P*), which is the probability of recapture of a surviving individual (Lebreton et al. 1992; Letcher et al. 2002; Letcher and Horton 2008). Apparent survival probability is the product of the survival probability and the probability of the fish remaining within in the area:


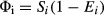
(1)

where Φ_*i*_ is the apparent survival probability between time *i*-1 and time *i*, *S*_*i*_ is the probability of survival between time *i*-1 and time *i* and *E*_*i*_ in the probability of emigration during the time between time *i*-1 and time *i*. Apparent survival probabilities are conservative survival estimates due to possible migration, and it is possible that mortality may therefore be less than 1-Φ. Apparent encounter probability is the product of the probability that an individual is available for capture and the true capture probability (P*):


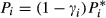
(2)

where *γ*_*i*_ is the probability of an individual being unavailable for capture at time *i*. Individuals can become unavailable for capture if they temporarily move out of the study area. In this study, apparent survival and apparent encounter probabilities between tagging in October and the first recapture event in spring were estimated. The second recapture event in spring allowed separate estimates for apparent survival and apparent encounter probabilities between October and the first recapture event to be obtained. As is typical for capture-mark-recapture methods, survival and encounter is confounded for the last sampling interval (between the first and second recapture event in spring) and separate estimates cannot be obtained for this last interval.

Cormack-Jolly-Seber (CJS) models were fitted using the package MARK (White and Burnham 1999), which uses a maximum-likelihood method. A range of models were compared, which according to model, allowed for variation in apparent survival probability and apparent capture probability according to site (ice-covered or ice-free) or size-class (parr or presmolt). Model selection was determined using the Akaike Information Criterion (AIC), a measure of the goodness of fit that includes a penalty that increases with number of parameters.

### Condition factor, specific energy, and fish movement

Condition factor, specific energy content and fish movements were quantified to aid interpretation of site-dependent and size class-dependent differences in apparent survival probability. Measurements of condition factor were missed on some individuals due to error. Condition factor (body mass[g] × 10^5^/body length[mm]^3^) was chosen as a metric of the relative well-being of the fish because it was possible to obtain without sacrificing the individual, thus enabling direct comparison of condition factor of an individual at release and recapture. To evaluate the validity of this, the relationship between condition factor and specific energy (J g^−1^) was determined using juveniles sampled pre and post winter at both sites, and during the winter at the upstream site (Table 2). Condition factor and specific energy were measured multiple times throughout winter at the ice-free site to determine if changes in these metrics occurred in a specific part of winter. It was not possible to measure condition factor and specific energy throughout winter in the ice-covered site due to the presence of ice.

**Table 2:** Dates and sample sizes used for establishing relationship between condition factor and specific energy of Atlantic salmon juveniles in the River Alta

Site	Date	Number of parr captured	Number of presmolts captured
Ice-covered	14 October 2004	50	50
Ice-covered	11 May 2005	147	150
Ice-free	14 October 2004	29	39
Ice-free	16 November 2004	37	30
Ice-free	22 Feburary 2005	18	13
Ice-free	3 April 2005	23	18
Ice-free	20 April 2005	33	33
Ice-free	8 May 2005	40	27

Specific energy was estimated from a regression relationship between the dry- to fresh-mass (dry-mass percentage) and specific energy (see Hartman and Brandt 1995) using parameters for juvenile Atlantic salmon from the River Alta established by Finstad et al. (2004b):


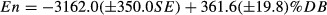
(3)

where *En* is the specific energy of the individual (J g^−1^), and%*DB* is the percentage dry body mass of the individual. To determine water content of the fish, the carcass, including intestines with fat deposits, but without stomach contents, was dried in a drying cabinet (70°C) until the weight stabilized. The relationship between individual condition factor and specific energy for each site and date was determined using Pearson's correlation.

Distances moved between release and first recapture were calculated from GPS measurements at release and recapture. Measurements of distance moved were missed on some individuals due to error. Absolute distance moved for tagged and recaptured individuals according to site and size class was compared using Mann–Whitney *U*-tests.

## Results

### Overwinter apparent survival probability and apparent encounter probability

**Figure 4:**
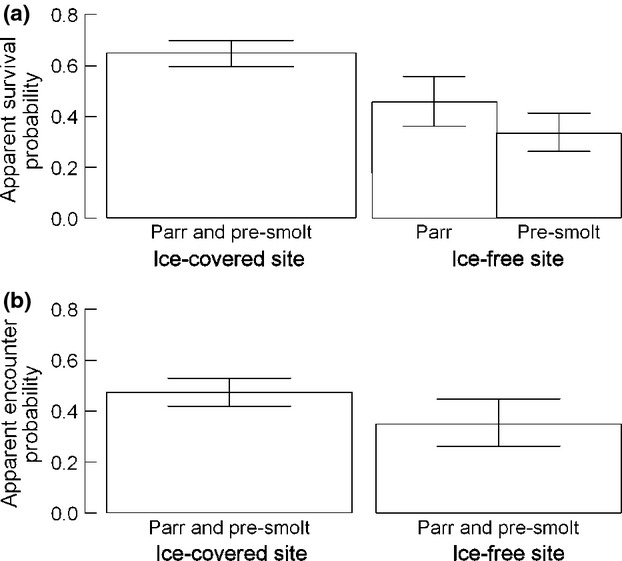
Estimates of (a) apparent survival probabilities and (b) apparent encounter probabilities of Atlantic salmon parr and presmolts in the ice-covered and ice-free sites from the optimal CJS model. Whiskers extend ± 2 SE. Apparent capture probability for parr and presmolts in the ice-free site in the optimal CJS model were equivalent.

The optimal model in terms of AIC had apparent survival probability varying according to size-class in the ice-free site, but not in the ice-covered site, and apparent encounter probabilities varying according to site, but not size-class (model No. 8 in Table 3). For the optimal model, the apparent survival probability was greater in the ice-covered site (Φ = 0.64 for both parr and presmolts) than in the ice-free site (Fig 4). In the ice-free site, presmolts had a lower apparent survival probability (Φ = 0.33) than parr (Φ = 0.45). The apparent encounter probability was greater in the ice-covered site (*P* = 0.47) than in the ice-free site (*P* = 0.35). The model with second lowest AIC (model No. 7 in Table 3) also showed a similar pattern with greater apparent survival probability in the ice-covered site (Φ = 0.67 for parr and Φ = 0.63 for presmolts) than in the ice-free site (Φ = 0.40 for parr and Φ = 0.40 for pre-smolts). The model with the third lowest AIC (model No. 5 in Table 3) also showed a similar pattern (Φ = 0.69 for parr and Φ = 0.65 for presmolts in the ice-covered site and Φ = 0.39 for parr and Φ = 0.28 for presmolts in the ice-free site). Thus, the choice among three models with fairly similar AIC values did not jeopardize our main finding of lower apparent survival in the ice-free site.

**Table 3:** Model of apparent survival probability (Φ) over the winter of 2004/2005 and apparent encounter probability (*P*), showing number of parameters (No. Par), degrees of freedom (df) and Akaike Information Criterion (AIC)

No.	Model	Description	No. Par	df	AIC
1	Φ*P*	Φ & *P* are same between sites and same between size classes	2	10	4845.6
4	Φ_site_*P*	Φ varies according to site	3	9	4632.0
2	Φ*P*_site_	*P* varies according to site	3	9	4647.2
3	Φ_site_*P*_site_	Φ & *P* vary according to site	4	8	4628.8
5	Φ_site,size_*P*	Φ varies according to site & size class	5	7	4624.0
6	Φ*P*_site,size_	P varies according to size and site	5	7	4636.2
7	Φ_site,size_*P*_site,size_	Φ & *P* vary according to site and size class	8	5	4622.5
8	Φ_site,size(Ice-free)_*P*_site_	Φ varies according to size class in ice-free site	5	7	4620.8
		Φ varies according to site			
		*P* varies according to site			

### Condition factor, specific energy and movements

**Figure 5:**
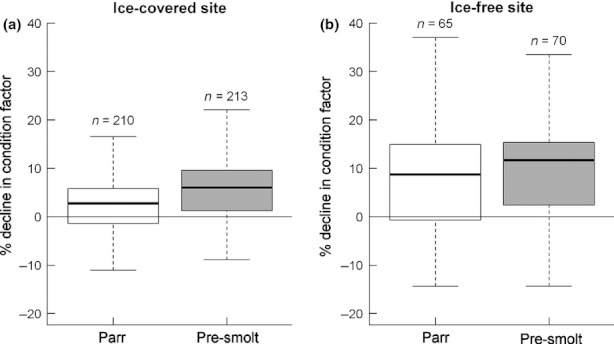
Percentage decline in condition factor between mark and first recapture: (a) ice-covered site; (b) ice-free site. White boxes show parr; gray boxes show presmolts. The number of individuals recaptured for which condition factor was measured is shown above each box. Thick horizontal line = median; the box bounds the first and third quartiles; whiskers = all values outside box within 1.5 × interquartile range.

**Figure 6:**
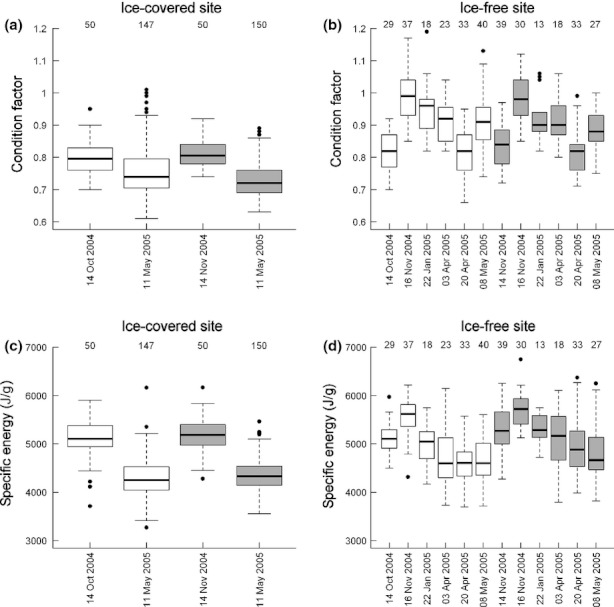
Condition factor and specific energy of sacrificed individuals: (a) condition factor at ice-covered site; (b) condition factor at ice-free; (c) specific energy at ice-covered site; (d) specific energy at ice-free site. White boxes show parr; gray boxes show presmolts. The number of individuals in each sample is shown above each box. Thick horizontal line = median; the box bounds the first and third quartiles; whiskers = all values outside box within 1.5 × interquartile range.

Condition factor declined between mark and recapture for both size classes at both sites (Mann–Whitney *U*-tests: ice-covered site, parr *P* < 0.001, presmolts *P* < 0.001; ice-free site, parr *P* = 0.005, presmolts *P* < 0.001) (Fig 5). The decline was greater in the ice-free site than the ice-covered site: medians of 2.7% and 8.7% for parr in ice-covered and ice-free sites, respectively (Mann–Whitney *U*-test, *P* < 0.001); medians 6.0% and 11.7 for presmolts in the ice-covered and ice-free site, respectively (Mann–Whitney *U*-test, *P* < 0.001). Condition factor and specific energy in samples collected post winter were less than those collected prewinter in the ice-covered site. At the ice-free site (which could be sampled throughout winter due to lack of ice cover), condition factor and specific energy showed a fairly consistent decline throughout winter (Fig 6). Condition factor was significantly correlated with specific energy for both sacrificed parr (Pearson's *r* = 0.52, *P* < 0.001) and presmolts (*r* = 0.69, *P* < 0.001). Given this, the condition factors of groups at mark and first recapture can be considered to be a reasonable indication of their relative energy content.

**Figure 7:**
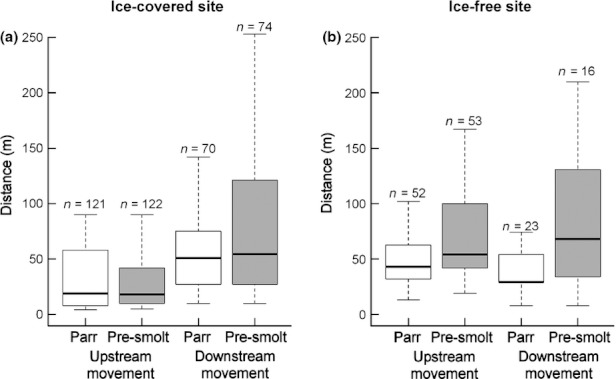
Distance moved between release and first recapture: (a) ice-covered site; (b) ice-free site. The number of individuals recaptured for each recapture location is shown above each box. White boxes show parr; gray boxes show presmolts. Thick horizontal line = median; the box bounds the first and third quartiles; whiskers = all values outside box within 1.5 × interquartile range.

Distances moved between mark and recapture were generally short, and in both upstream and downstream directions. No individuals tagged in the ice-free site were captured in the ice-covered site. In the ice-covered site, parr moved a similar distance to presmolts (Mann–Whitney *U*-test, *P* = 0.77) (Table 4). In the ice-free site, presmolts moved a significantly greater distance than parr (Mann–Whitney *U*-test, *P* < 0.001) between release and first recapture. Thirteen percent of first presmolt recaptures in the ice-free site were at a distance of greater than 200 m from release, whereas the corresponding figure for first parr recaptures was only 2.7% (Table 4). No significant difference existed between the sites in terms of absolute difference moved by parr (Mann-Whiney *U*-test, *P* = 0.10), but presmolts moved significantly further in the ice-free site than the ice-covered site (*P* < 0.001). There was a tendency to upstream movement at both sites (Table 4). However, in the ice-covered site, individuals that did move downstream tended to move further than those that moved upstream (Fig 7).

**Table 4:** Movements between release and first recapture according to site and size-class. Metrics shown are median and maximum distance moved, percentage of recaptures at a distance of >200 m from release and percentage of recaptures that were downstream

		Distance moved (m)		% of recaptures	
			
Site	Size-class	Median	Maximum	>200 m	Downstream
Ice-covered	Parr	38	411	6.8	36.7
	Presmolt	31	768	8.2	37.8
Ice-free	Parr	37	490	2.7	39.7
	Presmolt	54	1532	13.0	23.2

## Discussion

Apparent survival probability was lower in the ice-free than the ice-covered site, according to our hypothesis. However, whereas both size-classes had equivalent apparent survival in the ice-covered site, that of presmolts was lower than that of parr in the ice-free site. Apparent survival depends on both mortality and emigration; hence, our results suggest a real survival minimum of 66% (ice-covered site, both size groups), 45% (ice-free site, parr) and 33% (ice-free site, presmolts). The higher mortality in the ice-free site is supported by previous observations of abundances at the two sites. Both sites are fully recruited and have similar 1 + juvenile abundances, but in the ice-free site juvenile abundances decline faster with increasing age from 1 + to 4 + age groups and presmolt density is approximately half that of the ice-covered site (Ugedal et al. 2008). Moreover, the higher mortality and subsequently lower smolt production in the ice-free section of the river is also supported by a lower presmolt density, being approximately two to four times higher in the ice-covered site (Ugedal et al. 2008). As a result, the relative catch of returning adult fish in the ice-free upper part of the river has declined to approximately one quarter of catches in the middle part of the river compared with when the upper section was ice covered before the hydropower regulation ( Ugedal et al. 2008).

The lower survival rate of the larger size class does not concur with the majority of the literature, which has shown larger individuals having higher survival rates, both in experimental studies (Oliver et al. 1979; Post and Evans 1989; Johnson and Evans 1991) and in field studies (Post and Evans 1989; Miranda and Hubbard 1994). However, a reverse size-dependence has been observed in some studies. Connolly and Petersen (2003) found higher survival for smaller individuals, Carlson et al. (2008) found higher survival for smaller individuals in some cases and Letcher et al. (2002) found higher survival rates in 0 + than 1 + juveniles of Atlantic salmon – strengthening our assertion that this finding is biologically meaningful.

The first recapture survey of tagged fish was conducted 1 month earlier in the ice-free site than in the ice-covered site (starting date of 1 April and 1 May, respectively) due to operational reasons. Therefore, sampled individuals in the ice-free site were not experiencing mortality during April, a period that previous studies have shown to be critical for survival (Finstad et al. 2004b). Hence, it is probable that survival in the ice-free site would have been even lower had the first recapture taken place a month later, strengthening our assertion that mortality in the ice-free site was greater. Furthermore, apparent encounter probability was lower in the ice-free site. If the apparent encounter probabilities were in fact equal between the two sites, the difference between them in terms of apparent survival would have been even larger. Thus, our results are strong indications of lower overwinter survival in the ice-free site than in the ice-covered site.

### Ice-covered versus ice-free site

The difference in apparent survival between the ice-covered and the ice-free site could potentially be due to site-specific characteristics affecting survival (such as stranding mortality, prey availability, or habitat suitability) and/or site-specific characteristics biasing the estimate of survival (such as how the presence/absence of ice influences salmon emigration out of the sites during winter). Stranding mortality due to sudden drops in water discharge downstream of the power station has been suggested as a reason for the reduced juvenile densities in this area after regulation in 1987, but improved routines for operation of the hydropower station have reduced this problem considerably (Ugedal et al. 2008) and there were no such sudden drops reported during the study period. A difference in prey availability during winter has been suggested, but this hypothesis was rejected by Johansen et al. (2010), who found no such difference between the two sites during winter. The sites were selected so that their habitat characteristics were similar in terms of substrate size, depth, width, and current flow conditions. Given this, it is reasonable to infer that, in the absence of differences in ice cover, they would have similar suitability in terms of sheltering over winter. Finally, differences between the two sites in terms of emigration may have potentially contributed to the differences in apparent survival. Studies, mainly in smaller rivers, have found that movements in juvenile Atlantic salmon are more extensive in late autumn and before freeze-up than during winter when ice cover is present (Enders et al. 2008; Linnansaari et al. 2009; Linnansaari and Cunjak 2010). Movements and redistribution of fish may increase during dynamic ice conditions during mid-winter in rivers that are not permanently covered with surface ice (Stickler et al. 2007). It is therefore conceivable that emigration from the ice-free site will have been greater. However, we found no significant differences in distance moved during winter for parr between the ice-free and ice-covered sites. Presmolts at the ice-free site moved a significantly greater distance than those at the ice-covered site, but the median distance moved were not dramatically different in the two areas (58 m in the ice-free site vs. 31 m in the ice-covered site). At recapture, we extended the distance searched to 400 m below and 200 m above the area the fish were released, making it likely that we would have detected large differences in movement between the two areas. In both sites, median distances moved from release until recapture were small, suggesting that the habitats were suitable for over winter residence. Given that there was probably not a site-specific characteristic affecting survival or the bias of the survival estimate, the most plausible explanation is that the lack of surface ice cover caused the lower survival.

The lower survival in the ice-free site is consistent with the observation that, between release in mid-October and recapture in spring, the decline in condition factor was greater in the ice-free than in the ice-covered site. Given that there was a significant relationship between condition factor and specific energy, it can be inferred that there was a greater decrease in specific energy in individuals in the ice-free site. Hence, our results indicate that the lower survival at the ice-free site could have been linked to the lower energy status of the individuals. This is in accordance with previous studies, as both Finstad et al. (2004a) and Næsje et al. (2006) have identified high rates of energy depletion in the ice-free site, with Finstad et al. (2004a) showing evidence of energy dependent mortality based on a comparison of the population somatic energy frequency distributions between sample occasions supported by energetic modeling. It has been shown in laboratory studies that Atlantic salmon juveniles originating from rivers that are usually ice covered for several months during the winter may be adapted to ice-covered conditions, and show an increased activity level, reduced growth and higher energy loss during ice-free conditions (Finstad et al. 2004a; Finstad and Forseth 2006). Hence, reduced ice cover during winter, resulting in increased light levels, may influence juvenile salmon behavior such that there is increased activity, with consequent negative effects on energy budgets and survival.

Additionally, reduced ice cover may increase the predation risk by birds and mammals. Bird predators such as goosander (*Mergus merganser*), red-breasted merganser (*M. serrator*) and seagulls (*Larus* spp.) found in this area during summer are usually absent during winter (NINA, unpubl. data), but the mammalian predators American mink (*Neovison vison*) and otter (*Lutra lutra*) may occur. The most likely fish predator occurring on these stretches is large-grown brown trout (*Salmo trutta*). However, the general predation level on Atlantic salmon juveniles in this area during winter remains unknown.

### Parr versus pre-Smolt

Parr did not have a lower apparent survival than presmolts, contrary to our hypothesis. The effect of size on apparent survival differed with ice cover condition, with equal apparent survival of both size classes in the ice-covered site, and presmolts actually having lower apparent survival than parr in the ice-free site. An energetic based difference in mortality cannot be ruled out as presmolts had a larger decline in condition factor in the ice-free site compared to the ice-covered site. The critical body energy level for winter survival of juvenile salmon in the River Alta is approximately 4400–4800 J g^−1^ (Finstad et al. 2004b), and the mean energy level of both parr and presmolts in the ice-free site in the spring were near to critically low. Salmon juveniles cannot rely on stored energy alone to survive the winter and depend on acquiring food (Berg and Bremset 1998). Feeding during winter depends on the energy status of the fish and is assumed to be tailored to meet the long-term energy demands (Bull et al. 1996, Finstad et al. 2010). Larger individuals have a higher absolute energy demand relatively to smaller individuals (Peters 1983; Persson 1985). As a potential explanation of the lower apparent survival for presmolts in the ice-free sites compared with parr, it may therefore be suggested that larger fish increase activity levels in order to retain an energy intake and therefore suffer increased mortality.

The difference between the parr and presmolt groups is not only a size difference, but is also probably physiological. The presmolt group has likely started the smolting process in April and May, although they do not enter the sea before the end of June and beginning of July in this river (Davidsen et al. 2009). Smolting is an adaptive specialization occurring in freshwater as a preparation for the marine feeding migration, involving complex behavioral, morphological, energetic and other physiological changes (McCormick et al. 1998). The smolt stage is generally regarded as more sensitive than other life stages, which may explain the poorer performance of the pre-smolts than parr in this study, despite their larger body size. The smolting process is to a large extent regulated by photoperiod (McCormick et al. 1998). We might speculate that in a river where Atlantic salmon are adapted to ice cover during the winter and early spring, the natural smolting process and its timing might be disturbed by the lack of ice cover. This may cause additional stress, and possibly increase mortality in fish with already low energy levels, and thus be a plausible explanation of the lower survival of presmolts in the ice-free than the ice-covered site.

In the ice-free site, recaptured presmolts moved further than recaptured parr, but the greater distances moved were not only in a downstream direction, suggesting that these movements were not associated with an early downstream migration connected to the smolt run. It is possible that the greater movements of presmolts resulted from them seeking more appropriate habitat. During winter, juvenile salmon become nocturnal and seek refuge during the day to avoid diurnal predators (Valdimarsson and Metcalfe 1998). Ice cover can act as a refuge such that shelter in stream bed interstitial spaces is more important in the absence of ice cover (Linnansaari et al. 2009) and larger fish are more dependent on shelters (Finstad et al. 2007). Thus, the contrasting effects of ice cover on apparent survival of parr and presmolts could result from higher dependence on substrate shelter in ice-free areas, more severely affecting the larger fish. This may act either through (1) higher emigration of presmolts from ice-free areas (that is, apparent survival was less than the real survival) caused by increased shelter demand of larger individuals, or (2) increased direct mortality due to increased difficulty in finding shelter in larger individuals, or a combination of these two factors. Hence, it may be that our result somewhat overestimate the difference in survival between parr and presmolt size-classes in the ice-free site.

The Arctic Climate Impact Assessment (ACIA 2006) has concluded that increased winter survival resulting from future climate change may contribute to increased biomass in the Arctic in many fishes. However, this report also emphasizes ecosystem-specificity. The findings of our field study support this idea of ecosystem-specificity. At least within large rivers not subject to anchor ice, the likely reduction in winter surface ice cover caused by future climate change may have a negative effect on juvenile salmon survival during winter, and particularly for presmolts. Winter survival is only one of the phenomena that may affect salmon abundance in the arctic in future climates. Survival of adults at sea may be improved due to greater near-shore productivity and increased sea temperature, and compensate, at least partly, for reduced winter survival. Hence, our findings cannot be used to support the inference that climate change will inevitably result in reduced population abundance in large rivers with depleted ice cover, but they do suggest that at least the age distribution of the juvenile population may become more skewed to being composed of larger proportions of younger individuals.
